# Mechanism of Cooperative Degradation of Gum Arabic Arabinogalactan Protein by Bifidobacterium longum Surface Enzymes

**DOI:** 10.1128/aem.02187-21

**Published:** 2022-03-22

**Authors:** Yuki Sasaki, Masahiro Komeno, Akihiro Ishiwata, Ayako Horigome, Toshitaka Odamaki, Jin-Zhong Xiao, Katsunori Tanaka, Yukishige Ito, Kanefumi Kitahara, Hisashi Ashida, Kiyotaka Fujita

**Affiliations:** a The United Graduate School of Agricultural Sciences, Kagoshima University, Kagoshima, Kagoshima, Japan; b Graduate School of Biology-Oriented Science and Technology, Kindai University, Kinokawa, Wakayama, Japan; c RIKEN Cluster for Pioneering Research, Wako, Saitama, Japan; d Next Generation Science Institute, Morinaga Milk Industry Co., Ltd., Zama, Kanagawa, Japan; e Department of Chemical Science and Engineering, Tokyo Institute of Technology, Meguro, Tokyo, Japan; f Graduate School of Science, Osaka University, Toyonaka, Osaka, Japan; g Faculty of Agriculture, Kagoshima University, Kagoshima, Kagoshima, Japan; h Faculty of Biology-Oriented Science and Technology, Kindai University, Kinokawa, Wakayama, Japan; Kyoto University

**Keywords:** arabinofuranosidase, arabinogalactan protein, *Bacteroides*, bifidobacteria, glycosidase, gum arabic, prebiotics

## Abstract

Gum arabic is an arabinogalactan protein (AGP) that is effective as a prebiotic for the growth of bifidobacteria in the human intestine. We recently identified a key enzyme in the glycoside hydrolase (GH) family 39, 3-*O*-α-d-galactosyl-α-l-arabinofuranosidase (GAfase), for the assimilation of gum arabic AGP in Bifidobacterium longum subsp. *longum*. The enzyme released α-d-Gal*p*-(1→3)-l-Ara and β-l-Ara*p*-(1→3)-l-Ara from gum arabic AGP and facilitated the action of other enzymes for degrading the AGP backbone and modified sugar. In this study, we identified an α-l-arabinofuranosidase (BlArafE; encoded by BLLJ_1850), a multidomain enzyme with both GH43_22 and GH43_34 catalytic domains, as a critical enzyme for the degradation of modified α-l-arabinofuranosides in gum arabic AGP. Site-directed mutagenesis approaches revealed that the α1,3/α1,4-Ara*f* double-substituted gum arabic AGP side chain was initially degraded by the GH43_22 domain and subsequently cleaved by the GH43_34 domain to release α1,3-Ara*f* and α1,4-Ara*f* residues, respectively. Furthermore, we revealed that a tetrasaccharide, α-l-Rha*p*-(1→4)-β-d-Glc*p*A-(1→6)-β-d-Gal*p*-(1→6)-d-Gal, was a limited degradative oligosaccharide in the gum arabic AGP fermentation of B. longum subsp. *longum* JCM7052. The oligosaccharide was produced from gum arabic AGP by the cooperative action of the three cell surface-anchoring enzymes, GAfase, exo-β1,3-galactanase (Bl1,3Gal), and BlArafE, on B. longum subsp. *longum* JCM7052. Furthermore, the tetrasaccharide was utilized by the commensal bacteria.

**IMPORTANCE** Terminal galactose residues of the side chain of gum arabic arabinogalactan protein (AGP) are mainly substituted by α1,3/α1,4-linked Ara*f* and β1,6-linked α-l-Rha*p*-(1→4)-β-d-Glc*p*A residues. This study found a multidomain BlArafE with GH43_22 and GH43_34 catalytic domains showing cooperative action for degrading α1,3/α1,4-linked Ara*f* of the side chain of gum arabic AGP. In particular, the GH43_34 domain of BlArafE was a novel α-l-arabinofuranosidase for cleaving the α1,4-Ara*f* linkage of terminal galactose. α-l-Rha*p*-(1→4)-β-d-Glc*p*A-(1→6)-β-d-Gal*p*-(1→6)-d-Gal tetrasaccharide was released from gum arabic AGP by the cooperative action of GAfase, GH43_24 exo-β-1,3-galactanase (Bl1,3Gal), and BlArafE and remained after B. longum subsp. *longum* JCM7052 culture. Furthermore, *in vitro* assimilation test of the remaining oligosaccharide using *Bacteroides* species revealed that cross-feeding may occur from bifidobacteria to other taxonomic groups in the gut.

## INTRODUCTION

**FIG 1:**
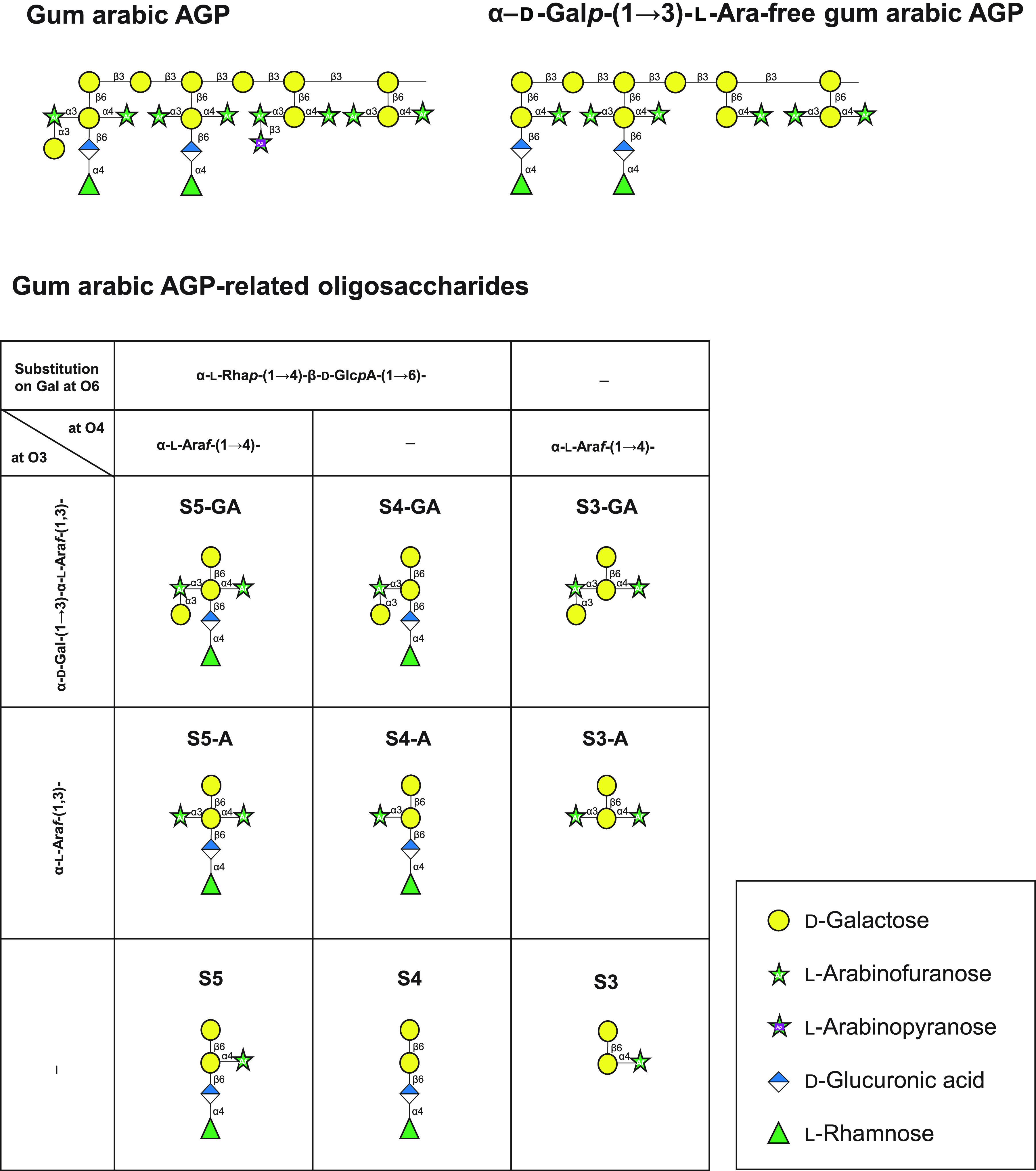
Schematic structures of gum arabic AGP, α-d-Gal*p*-(1→3)-l-Ara-free gum arabic AGP, and gum arabic AGP-related oligosaccharides used in this study.

Arabinogalactan protein (AGP) is one of the highly complex glycoprotein components of plant cell walls. It is widely found in edible plant parts, such as vegetables, fruits, and cereals (1). The carbohydrate component of AGP is composed of type II arabinogalactan (AG) chains comprising a β1,3-galactan backbone chain with β1,6-galactan side chains. The side chain structure of AGPs varies among plants and organs. In particular, gum arabic is an AGP with complex sugar modifications at the branch (2) and is reported to act as a prebiotic without digestion by host enzymes and increases bifidobacteria levels in the large intestine (3, 4). In the gum arabic AGP, the terminal galactose residues of the side chain are mainly substituted by α1,3-linked Ara*f*, α-d-Gal*p*-(1→3)-l-Ara, or β-l-Ara*p*-(1→3)-l-Ara and α1,4-linked Ara*f* and β1,6-linked α-l-Rha*p*-(1→4)-d-Glc*p*A residues (Fig. 1) (5–8).

Recently, the mechanisms involved in the assimilation of gum arabic AGP by human gut microbiota have been revealed for *Bacteroides* and bifidobacteria. Regarding *Bacteroides*, Cartmell et al. revealed the function of polysaccharide utilization loci involved in the degradation of gum arabic AGP in B. thetaiotaomicron and B. cellulosilyticus and reported that the cell surface endo-β1,3-galactanase was a key enzyme for assimilating gum arabic AGP (7). They also characterized many other related enzymes for trimming modified sugar in the periplasm. For the degradation of α-l-Ara*f*, α-l-arabinofuranosidase (BT3675) was characterized to hydrolyze α1,3-Ara*f* linkage but not α1,4-Ara*f* linkage, but α1,4-Ara*f*-specific α-l-arabinofuranosidase has not been detected yet. In bifidobacteria, we found a key enzyme in the glycoside hydrolase (GH) family 39, namely, 3-*O*-α-d-galactosyl-α-l-arabinofuranosidase (GAfase), for the assimilation of gum arabic AGP in Bifidobacterium longum subsp. *longum* JCM7052 (9). The enzyme released α-d-Gal*p*-(1→3)-l-Ara and β-l-Ara*p*-(1→3)-l-Ara from gum arabic AGP. We also found that GAfase released disaccharides as a carbon source and promoted the action of other type II AG-degrading enzymes. However, the combination of these type II AG degradative enzymes (GAfase, exo-β1,3-galactanase [Bl1,3Gal], and α-l-arabinofuranosidase [BlArafA]) used in the previous study could not release the same amount of l-arabinose as that released by the bacterial cell fraction used. Therefore, it remains unclear which α-l-arabinofuranosidases mainly act on α1,4-Ara*f* linkage of gum arabic AGP.

Based on the Carbohydrate-Active enZymes (CAZy) database, B. longum subsp. *longum* JCM1217 encodes nine α-l-arabinofuranosidase candidates, of which six are cell surface-anchoring GH43s and three are intracellular GH51s. In GH43s, HypAA (BLLJ_0213) contained the GH43 subfamily 29 (GH43_29) domain and was reported to be an α1,3-Ara*f*-specific α-l-arabinofuranosidase for degrading α-l-Ara*f*-(1→3)-β-l-Ara*f*-(1→2)-β-l-Ara*f*-(1→2)-β-l-Ara*f*-Hyp (10). BLLJ_1850 to BLLJ_1854 are arranged in tandem in the gene cluster, and we previously reported that BlArafA (BLLJ_1854) contained the GH43 subfamily 22 (GH43_22) domain acting on the α1,3-Ara*f* residue of larch AGP (11). BlArafB (BLLJ_1853) contained the GH43_22 domain, which hydrolyzes the α1,5-Ara*f* linkage of arabinan backbone, and BlArafC (BLLJ_1852) contained the GH43 subfamily 27 (GH43_27) domain, which hydrolyzes the α1,2- and α1,3-Ara*f* linkages on arabinan side chains (12). Recently, it was discovered that BlArafD (BLLJ_1851) and BlArafE (BLLJ_1850) cooperatively degrade arabinoxylan (M. Komeno, Y. Yoshihara, J. Kawasaki, W. Nabeshima, K. Maeda, Y. Sasaki, K. Fujita, H. Ashida, unpublished data). BlArafD (BLLJ_1851) contained GH43_UC and GH43_26 domains. GH43_UC acts on the α1,2-Ara*f* linkages of the α1,2- and α1,3-Ara*f* doubly substituted arabinoxylan, whereas GH43_26 acts on the arabinan backbone. BlArafE (BLLJ_1850) contains GH43_22 and GH43_34 domains, wherein GH43_22 acts on the α1,3-Ara*f* linkage in arabinoxylan. However, the specificity of GH43_34 remains unclear.

**FIG 2:**
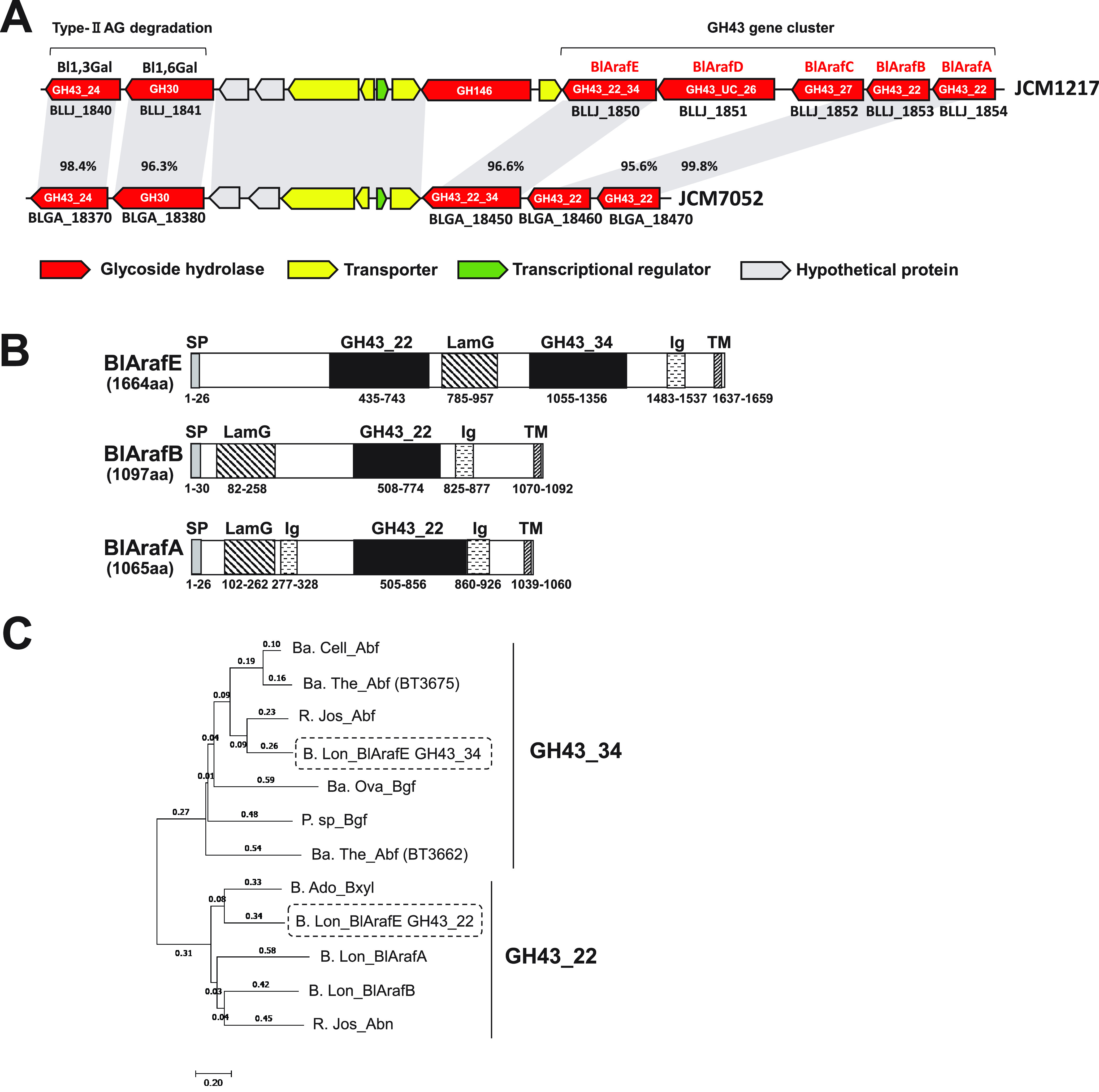
Structural features of the α-l-arabinofuranosidase candidates. (A) Gene clusters in B. longum subsp. *longum* JCM1217 and JCM7052 involved in the degradation of type II AG. The arrowheads (with names below) indicate genes annotated in the Kyoto Encyclopedia of Genes and Genomes database. The glycoside hydrolases (GHs) in the Carbohydrate-Active enZymes (CAZy) database are shown inside the red arrowheads. Light gray bars indicate orthologous regions (>95% identity). (B) Domain structures of BlArafE, BlArafB, and BlArafA. Domain structures were predicted using SignalP5.0 (http://www.cbs.dtu.dk/services/SignalP/) and InterPro (https://www.ebi.ac.uk/interpro/) servers. The domains are indicated as follows: SP, signal peptide; LamG, laminin G; Ig, bacterial Ig-like domain; TM, transmembrane region. (C) Phylogenetic tree of GH43_34 and GH43_22 domains of BlArafE. The phylogenetic tree was constructed using the neighbor-joining method and the aligned sequences. For constructing the tree, the program MUSCLE was implemented in MEGA7 software. GH43_34 and GH43_22 domains of BlArafE are enclosed in the dashed-line box. The characterized enzymatic activities or protein names are shown alongside the abbreviated names of the organisms as follows: Ba. Cell_Abf, B. cellulosilyticus α-l-arabinofuranosidase (GenPept accession no. ALJ58905.1); Ba. The_Abf (BT3675), B. thetaiotaomicron α-l-arabinofuranosidase encoded by BT3675 (GenPept accession no. AAO78780.1); R. Jos_Abf, Ruminiclostridium josui exo-α1,5-arabinofuranosidase (GenPept accession no. BBA94052.1); Ba. Ova_Bgf, B. ovatus β-d-galactofuranosidase (GenPept accession no. ALJ48250.1); P. sp_Bgf, *Paenibacillus* sp. strain β-d-galactofuranosidase (ACS99115.1); Ba. The_Abf (BT3662), B. thetaiotaomicron α-l-arabinofuranosidase encoded by BT3662 (GenPept accession no. AAO78780.1); B. Ado_Bxyl, B. adolescentis β-xylosidase (GenPept accession no. BAF40308.1); B. Lon_BlArafA, B. longum subsp. *longum* JCM1217 BlArafA (GenPept accession no. BAJ67518.1); B. Lon_BlArafB, B. longum subsp. *longum* JCM1217 BlArafB (GenPept accession no. BAJ67517.1); and R. Jos_Abn, R. josui arabinanase (GenPept accession no. BBA94052.1).

The α-l-Ara*f* structure presented α1,2/1,3/1,5-linkages in some components of the plant cell wall (13), such as arabinan (14, 15), arabinoxylan (16), arabinoxyloglucan (17, 18), AG (6, 19, 20), hydroxyproline-linked β-l-arabinooligosaccharides (21), and rhamnogalacturonan (RG) II (22). Conversely, the α1,4-linkage of l-arabinose was found in the RG-II structure of pectin in the pyranose form (22), and to the best of our knowledge, the α1,4-Ara*f* structure was only found in gum arabic AGP among plant-derived glycans (8, 23). Hence, α1,4-Ara*f*-specific α-l-arabinofuranosidase has not been characterized so far in any organism due to this limited localization. The removal of the α1,3-linkage by GAfase was expected to remove the steric hindrance for an enzyme acting on the α1,4-Ara*f* linkage connected to the terminal galactose. Therefore, we tried to identify an α-l-arabinofuranosidase specific for α1,4-Ara*f* linkage. In our previous report, α-d-Gal*p*-(1→3)-l-Ara-free gum arabic AGP was fermented by B. longum subsp. *longum* JCM1217 and JCM7052 (9). The common α-l-arabinofuranosidase genes in the GH43 gene cluster found in the two strains were *BlArafA*, *BlArafB*, and *BlArafE* (Fig. 2A). Among them, we focused on the α-l-arabinofuranosidase candidate BlArafE, containing two catalytic domains, GH43_22 and GH43 subfamily 34 (GH43_34).

This study characterized each GH43_22 and GH43_34 domain of BlArafE that acted to α1,3/α1,4-Ara*f* continuously and determined the structure of the remaining oligosaccharide in gum arabic AGP fermentation of B. longum subsp. *longum* JCM7052. We also evaluated the ability to assimilate the limit product from B. longum in some *Bacteroides* species.

## RESULTS

### Structural features of α-l-arabinofuranosidase BlArafE.

B. longum subsp. *longum* JCM7052 possessed *BlArafE*, *BlArafB*, and *BlArafA* homologous genes with high sequence identity (>95%) but did not possess *BlArafC* and *BlArafD* homologous genes (Fig. 2A). *BlArafE*, *BlArafB*, and *BlArafA* produced cell surface-anchoring enzymes with a signal peptide at the N terminus and a transmembrane region at the C terminus and conserved GH43_22, laminin G, and bacterial Ig-like domains (Fig. 2B). BlArafE was a multidomain enzyme comprising two catalytic domains of α-l-arabinofuranosidase candidates, GH43_22 and GH43_34. The GH43_22 and GH43_34 domains of BlArafE were compared using a molecular phylogenetic tree with the previously characterized GH43 enzymes (Fig. 2C). The GH43_34 domain of BlArafE had the highest homology (57% identity) with exo-α1,5-arabinofuranosidase from Ruminiclostridium josui (24), and the GH43_22 domain had the highest homology (51% identity) with β-xylosidase from Bifidobacterium adolescentis (25). The GH43_22 domain of BlArafE showed 35% and 33% identity with BlArafA and BlArafB, respectively.

### Characterization of BlArafE.

**FIG 3:**
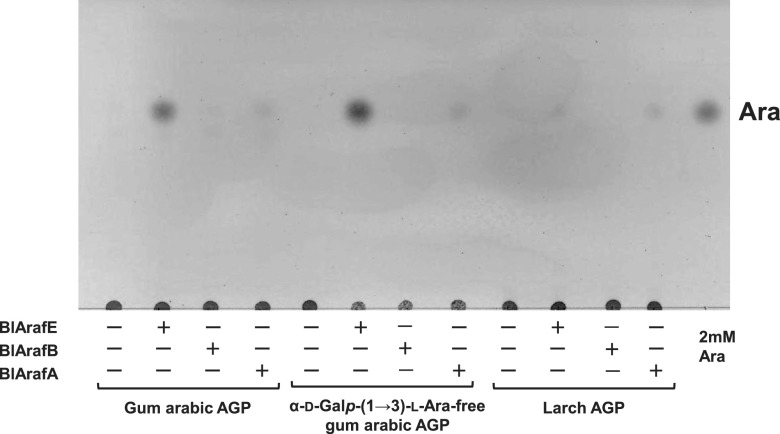
TLC analysis of the reaction products of α-l-arabinofuranosidases with AGPs. Gum arabic AGP, α-d-Gal*p*-(1→3)-l-Ara-free gum arabic AGP, and larch AGP were incubated with either BlArafE, BlArafB, or BlArafA at 37°C for 16 h.

**FIG 4:**
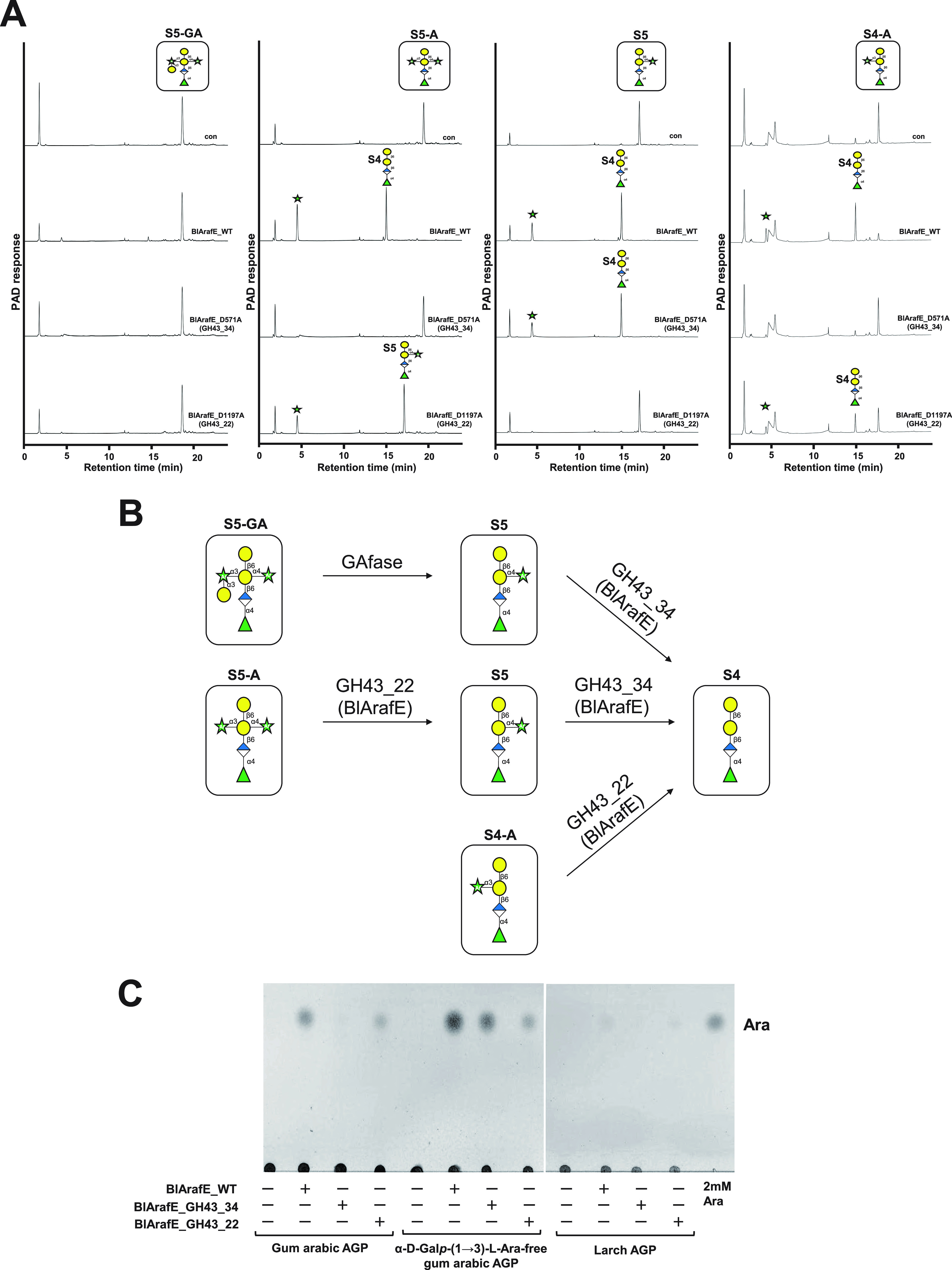
Substrate specificities of GH43_22 and GH 43_34 domains of BlArafE. (A) HPAEC-PAD analysis of the reaction products with gum arabic AGP-related oligosaccharides. S5-GA, S5-A, S5, and S4-A were incubated with either wild-type BlArafE (WT), D571A mutant BlArafE (GH 43_34 active form), or D1197A mutant BlArafE (GH 43_22 active form) at 37°C for 16 h. (B) Schematic model of the mode of action of GH43_22 and GH 43_34 domains of BlArafE. (C) TLC analysis of the reaction products with AGPs. Gum arabic AGP, α-d-Gal*p*-(1→3)-l-Ara-free gum arabic AGP, and larch AGP were incubated with either wild-type BlArafE, GH 43_34 active form BlArafE, or GH 43_22 active form BlArafE at 37°C for 16 h.

Recombinant BlArafE protein was designed without the N-terminal signal peptide (amino acids [aa] 1 to 26) and the C-terminal transmembrane region (aa 1637 to 1659) using the primers shown in Table 1. The recombinant molecule was highly expressed as a soluble protein at 25°C and was purified using a C-terminal His-tag. The purified recombinant BlArafE was found to migrate as a band with an apparent molecular mass of 175 kDa on sodium dodecyl sulfate-polyacrylamide gel electrophoresis (SDS-PAGE), corresponding to its calculated molecular mass (see Fig. S1 in the supplemental material). BlArafA and BlArafB were also expressed and purified according to the method described in previous studies (11, 12). Gum arabic AGP, larch AGP, and α-d-Gal*p*-(1→3)-l-Ara-free gum arabic AGP were treated with the recombinant enzymes, and the released sugars were analyzed by thin-layer chromatography (TLC) (Fig. 3). α-d-Gal*p*-(1→3)-l-Ara-free gum arabic AGP was prepared from gum arabic AGP by GAfase treatment, and the structural model is presented in Fig. 1. As a result, BlArafE and BlArafA released l-arabinose from all the tested polysaccharides, but BlArafB showed no activity. Notably, BlArafE released the largest amount of l-arabinose from gum arabic AGP and α-d-Gal*p*-(1→3)-l-Ara-free gum arabic AGP among the three enzymes. In the current study, several oligosaccharides constituting the side chains of gum arabic AGP were prepared (Fig. 1 and Fig. S2) to evaluate the substrate specificities of these enzymes in more detail, and each enzyme was incubated with oligosaccharides S5-GA, S5-A, S5, S4-A, S3-GA, S3-A, and S3 (Fig. 1). The released sugars were analyzed using high-performance anion-exchange chromatography with pulsed amperometric detection (HPAEC-PAD) (Fig. 4A and Fig. S3). As a result, we found that only BlArafE showed α1,4-Ara*f* cleavage activity on the oligosaccharide S5, but not BlArafB and BlArafA. Furthermore, BlArafE cleaved S5-A into S4 and L-arabinose, indicating that BlArafE can act on both α1,4-Ara*f* and α1,3-Ara*f*. Interestingly, when α1,3-Ara*f* in S5-A was capped with α1,3-Gal, such as S5-GA, BlArafE could not act on α1,4-Ara*f*, indicating that eliminating the α-d-Gal*p*-(1→3)-l-Ara or α-d-Gal*p* is essential for BlArafE activity. Conversely, BlArafA showed activity against α1,3-Ara*f* on S4-A but did not act on S5-A, in which β-d-Gal was double substituted with α-l-Ara*f* at O3 and O4. BlArafB could not hydrolyze any substrates with α-l-Ara*f* at O3 and O4. Similarly, we evaluated the enzyme reactivities for S3-GA, S3-A, and S3, in which β-d-Gal was not substituted by α-l-Rha*p*-(1→4)-β-d-Glc*p*A at O6 (Fig. S3). As a result, we found that BlArafE acted on S3-A and S3, suggesting that BlArafE exhibited activity regardless of whether α-l-Rha*p*-(1→4)-β-d-Glc*p*A was present or not.

**TABLE 1:** Primers used for plasmid construction and site-directed mutagenesis^*a*^

Primer name	Sequence of oligonucleotide primers
BLLJ_1850_for	5′-CCCAAGCTTGATACCACCGATTCATCGGCCGC-3′
BLLJ_1850_rev	5′-GTGCCGCTCGAGGGAGATGACGGCACCCGGCTTCTTG-3′
BLLJ_1850_D571A_for	5′-ACCACGATGATTAAGGCCGA-3′
BLLJ_1850_D571A_rev	5′-TGCGATAACGGACTTGAC-3′
BLLJ_1850_D1197A_for	5′-CCGTCCATCTTCACCGACC-3′
BLLJ_1850_D1197A_rev	5′-TGCGATGGCCTGTCCAAC-3′

a Positions of the mutated sequences are underlined.

### Functional analysis of each of the catalytic domains in BlArafE.

BlArafE conserved three critical putative acidic amino acid residues for catalytic reaction, “general acid,” “general base,” and “pKa modulator of general acid,” based on the previously characterized GH43 enzymes (26). Herein, the putative pKa modulator of general acid residues in BlArafE GH43_22 (Asp571) and BlArafE GH43_34 (Asp1197) were mutated to Ala to inactivate the respective domains. Similar to wild-type BlArafE, the mutants were expressed, and the purified recombinant BlArafE mutants were found to migrate as a single band with an apparent molecular mass of 175 kDa on SDS-PAGE, corresponding to their predicted molecular masses (data not shown). The oligosaccharide substrates (S5-GA, S5-A, S5, and S4-A) were used to evaluate the substrate specificity of GH43_22 and GH 43_34 domains. The BlArafE_D1197A mutant (GH43_22 active form) exhibited enzymatic activity toward α1,3-Ara*f* in α1,3- and α1,4-Ara*f* double-substituted structures in S5-A and single α1,3-substituted structures in S4-A, but could not act on α1,4-Ara*f* in S5 (Fig. 4A). S4-A was incompletely degraded by GH43_22 under the described conditions, and the active form of GH43_22 likely preferred double-substituted S5-A over single-substituted S4-A. Conversely, the BlArafE_D571A mutant (GH43_34 active form) acted on α1,4-Ara*f* in S5 and degraded it to S4, but it did not act on α1,3-Ara*f* in S4-A, which had a single substitution of α-l-Ara*f* at the O3 of the terminal Gal. The GH43_34 active form also showed no activity when β-d-Gal of the side chain was double substituted with α-l-Ara*f* at O3 and O4 in S5-A. S5-GA could not be hydrolyzed by either wild-type BlArafE or both mutants. In summary, the degradation of S5-A to S4 by BlArafE was first mediated via the digestion of α1,3-Ara*f* by GH43_22, followed by the digestion of α1,4-Ara*f* by GH43_34 (Fig. 4B).

Gum arabic AGP, α-d-Gal*p*-(1→3)-l-Ara-free gum arabic AGP, and larch AGP were treated with GH43_22 and GH43_34 active forms, providing insights into the structural model of gum arabic and larch AGPs. The GH43_22 active form released l-arabinose from gum arabic AGP (Fig. 4C), indicating that there are some α1,3-Ara*f* residues without α-d-Gal capping in gum arabic AGP (Fig. S4). Furthermore, the GH43_34 active form released many l-arabinose residues from α-d-Gal*p*-(1→3)-l-Ara-free gum arabic AGP but only a few from intact gum arabic AGP. These results indicated that S5-GA is one of the major components of the side chains of gum arabic AGP, rather than S5 or S3 structures with the single substitution of α-l-Ara*f* at O4, which was consistent with a previous study (7). Moreover, the GH43_34 active form could not release l-arabinose from larch AGP, indicating that there is no single-substituted α1,4-Ara*f* in larch AGP.

### *In vitro* assimilation test of gum arabic AGP using BlArafE carrier strains.

**FIG 5:**
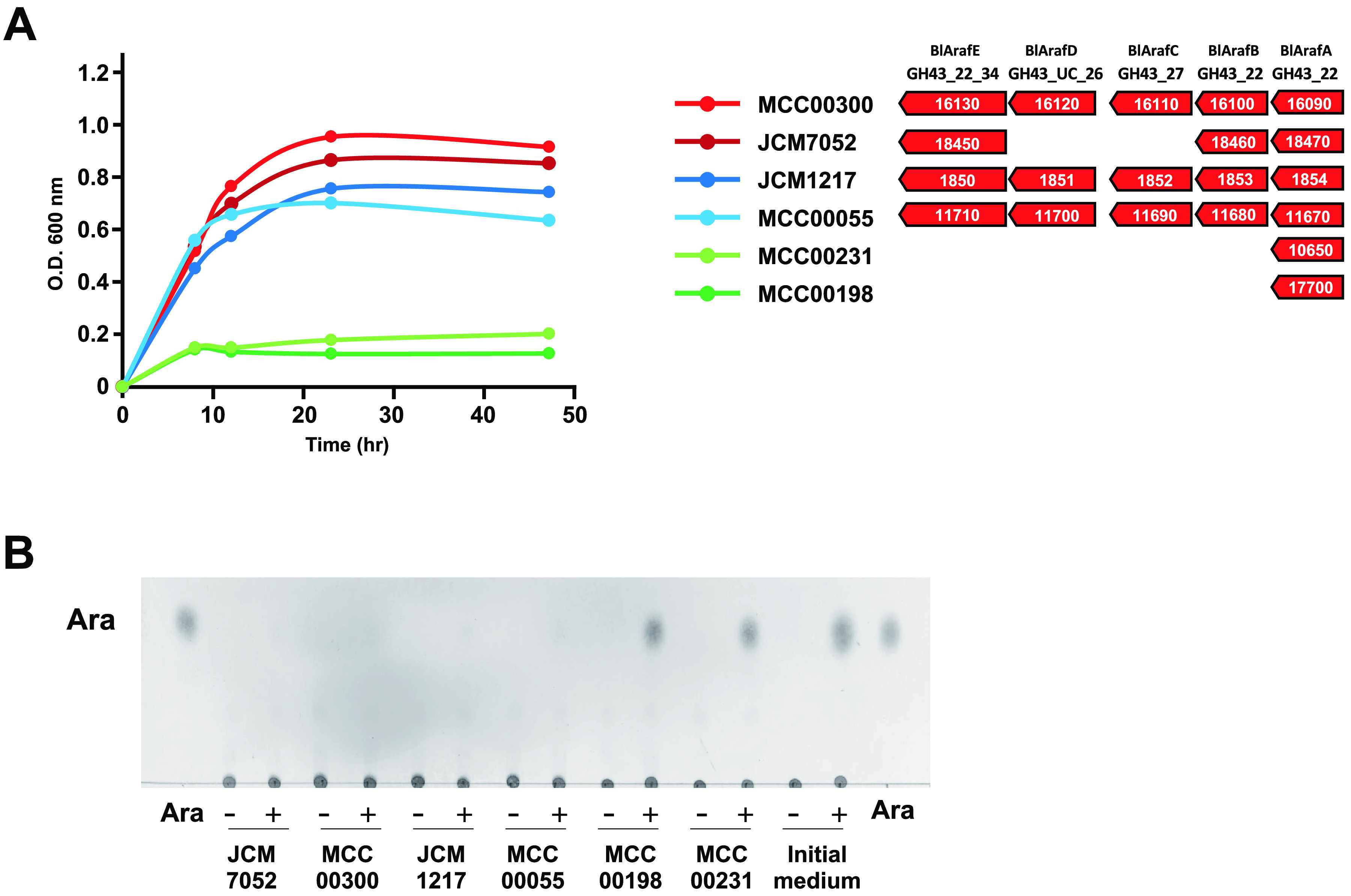
*In vitro* assimilation test performed using α-d-Gal*p*-(1→3)-l-Ara-free gum arabic AGP. (A) Growth profile of B. longum strains cultured in a medium containing α-d-Gal*p*-(1→3)-l-Ara-free gum arabic AGP (*n *= 2, cell culture biological replicates). The gene clusters of GH43 α-l-arabinofuranosidases in B. longum strains are shown in the figure. (B) Residual l-arabinose in α-d-Gal*p*-(1→3)-l-Ara-free gum arabic AGP after 48 h of culturing B. longum. TLC analysis of the reaction products by BlArafE with the ethanol precipitates of the initial medium or cultured media of JCM7052, MCC00300, JCM1217, MCC00055, MCC00198, and MCC00231 at 37°C for 16 h. Reaction products (+) and control samples without BlArafE (−) were analyzed by TLC.

The gene cluster of GH43 α-l-arabinofuranosidases (BLLJ_1850 to BLLJ_1854 homologs) has various conservation patterns in different strains of B. longum (12). The *BlArafE* homologous gene (identity ≧ 50%, coverage ≧ 50%) was conserved in 54.5% of B. longum subsp. *longum* MCC strains (67/123 strains) in the National Center for Biotechnology Information (NCBI) database, which was higher than GAfase (4.88%, 6/123 strains) (9). We performed an *in vitro* assimilation test using BlArafE carrier strains of B. longum and noncarrier strains in a medium containing α-d-Gal*p*-(1→3)-l-Ara-free gum arabic AGP as the sole carbon source (Fig. 5A). BlArafE carrier strains, B. longum subsp. *longum* JCM7052, MCC00300, JCM1217, and MCC00055, grew in the medium, whereas noncarrier strains, MCC00198 and MCC00231, exhibited no growth. Further, intact gum arabic AGP was only assimilated in the GAfase-carrier strains JCM7052 and MCC00300 (data not shown), which was in agreement with our previous study (9). Subsequently, BlArafE was incubated with the ethanol precipitate of the medium in order to examine whether l-arabinose remained to be attached to α-d-Gal*p*-(1→3)-l-Ara-free gum arabic AGP after culture. It was found that BlArafE released l-arabinose from the residual polysaccharides in noncarrier strains but not carrier strains (Fig. 5B). In general, B. longum strains are known to utilize l-arabinose as a carbohydrate source (27, 28). Therefore, these results suggested that BlArafE carrier strains utilized l-arabinose released from α-d-Gal*p*-(1→3)-l-Ara-free gum arabic AGP during their growth. GAfase noncarrier strains could not grow well on intact gum arabic AGP, even though these strains carried *BlArafE*. Hence, it is necessary to remove the steric hindrance of BlArafE by the action of GAfase to facilitate good growth.

### Cooperative degradation of gum arabic AGP.

**FIG 6:**
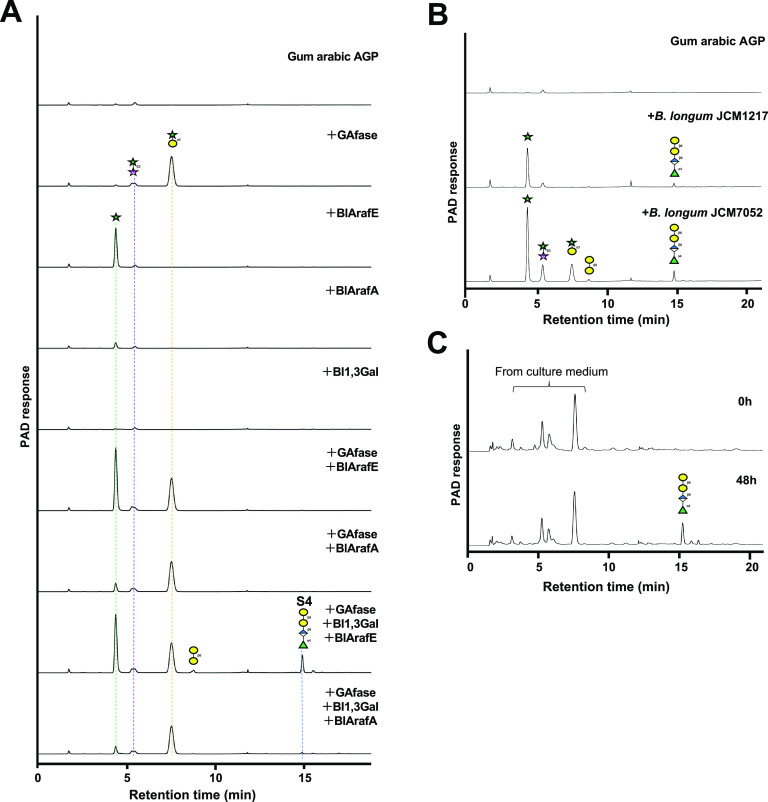
Cooperative degradation of gum arabic AGP by the cell surface-anchoring enzymes in B. longum. (A) HPAEC-PAD analysis of the combination reactions of type II AG degradative enzymes (GAfase, Bl1,3Gal, BlArafA, and BlArafE) with gum arabic AGP. Gum arabic AGP was incubated at 37°C for 24 h. Data from one representative experiment out of three independent experiments is shown. (B) HPAEC-PAD analysis of the reaction products by the bacterial cell fraction of B. longum subsp. *longum* JCM1217 or JCM7052 cells with gum arabic AGP. (C) HPAEC-PAD analysis of the initial medium (0 h) and cultured medium (48 h of B. longum subsp. *longum* JCM7052 culture) with 1% gum arabic AGP as the sole carbon source.

Gum arabic AGP was incubated with different combinations of GAfase, Bl1,3Gal, and α-l-arabinofuranosidases (BlArafE and BlArafA) to assess its cooperative degradation (Fig. 6A). The amount of l-arabinose released from gum arabic AGP incubated with the combination of GAfase and BlArafE was 1.64-fold greater than that released from gum arabic AGP incubated with BlArafE alone, indicating that GAfase could remove the steric hindrance and allowed BlArafE to act on α1,4-Ara*f* linkage. Furthermore, the peaks of l-arabinose, β-d-Gal-(1→6)-d-Gal, and S4 were detected by using the combination of GAfase, Bl1,3Gal, and BlArafE. The amounts of l-arabinose and S4 released by GAfase, Bl1,3Gal, and BlArafE were 10.5-fold and 13.6-fold higher, respectively, than those released by the combination of GAfase, Bl1,3Gal, and BlArafA instead of BlArafE. In addition, the peaks of the reaction product obtained by the combination of GAfase, Bl1,3Gal, and BlArafE were consistent with the reaction product obtained using B. longum subsp. *longum* JCM7052 cells as a crude enzyme (Fig. 6B). These results indicated that gum arabic AGP was degraded mainly by the action of three cell surface degradative enzymes (GAfase, Bl1,3Gal, and BlArafE) present in B. longum subsp. *longum* JCM7052. Furthermore, S4 remained in B. longum subsp. *longum* JCM7052 culture, but monosaccharides and disaccharides were not detected after 48 h. These results indicated that B. longum utilized monosaccharides and disaccharides, but not S4 (Fig. 6C).

### Structural determination of the limit product S4 and utilization by other commensal bacteria.

**FIG 7:**
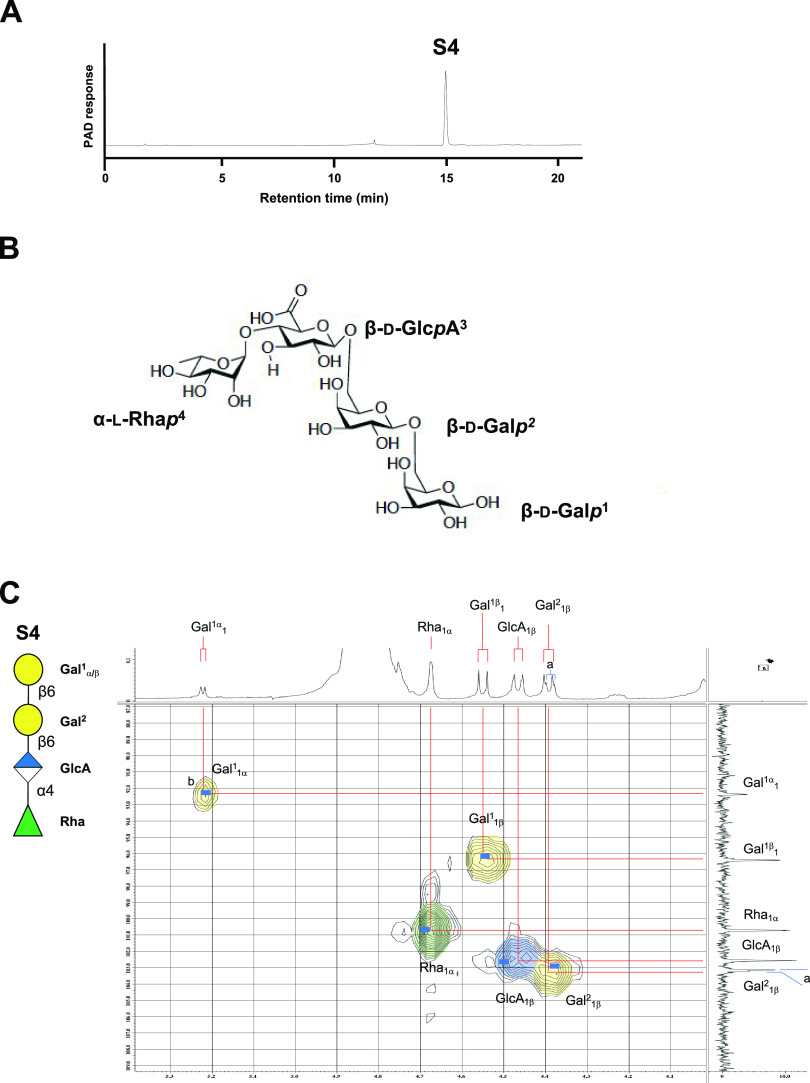
Structural analysis of S4. (A) HPAEC-PAD analysis of the purified S4. (B) Chemical structure of S4. (C) Expanded HMQC spectra around anomeric region measured at 400 MHz in D_2_O at 21°C. ^a^Difference between the anomers of Gal^1^ (α/β, approximately 1:2) slightly affected the conformation of Gal^2^ that was seen in Gal^2^_1β_. ^b^Assignment of the tetrasaccharide was compared to the HMQC data referenced at Gal^1^_1α_ indicated by blue bars, translated from the data reported by Cartmell et al. (7).

S4 was purified as a single peak from the limit product of the gum arabic AGP incubated with B. longum subsp. *longum* JCM7052 bacterial cells (Fig. 7A). Matrix-assisted laser desorption ionization–time of flight mass spectrometry (MALDI-TOF MS) of S4 revealed a high-intensity peak at 687.138 *m/z*, consistent with a sodium adduct of α-l-Rha*p*-(1→4)-β-d-Glc*p*A-(1→6)-β-d-Gal*p*-(1→6)-d-Gal (calculated for C_24_H_40_O_21_Na_1_ [M+Na]^+^ 687.196) (Fig. S5A). High-resolution electrospray ionization–time of flight mass spectrometry (ESI-TOF MS) of S4 revealed that the tetrasaccharide structure matched the elemental composition (ESI-TOF high-resolution MS [ESI-TOF HRMS] calculated for C_24_H_40_O_21_Na_1_ [M+Na]^+^ 687.1960, found 687.1958). We also identified the chemical structure of S4 (Fig. 7B) using nuclear magnetic resonance (NMR) analysis, including ^1^H, ^13^C, and heteronuclear multiple quantum correlation (HMQC) spectra (Fig. 7C and Table 2). ^1^H and ^13^C chemical shifts and coupling constants of S4 were α-l-Rha*p*-(1→4)-β-d-Glc*p*A-(1→6)-β-d-Gal*p*-(1→6)-d-Gal (Table 2), which matched with that determined by the previous reports (7). HMQC spectra showed that the overlap of anomeric regions was consistent with a previous report (7). In addition, the combination of NMR techniques, such as ^1^H-^1^H correlated spectroscopy (COSY), HMQC, and heteronuclear multiple-bond correlation (HMBC), and nondecoupled HMQC spectra of S4 further increased the validity of the chemical structure of S4 as the peaks assigned in Table 2 (Fig. S5B).

**TABLE 2:** Assignment of signal in ^1^H and ^13^C NMR spectra of S4 in D_2_O at 400 MHz at room temperature^*a*^

Unit	Value for:											
	H1 (^3^J_H–H_)	H2	H3	H4	H5	H6	C1 (^1^J_C–H_)	C2	C3	C4	C5	C6
β-d-Gal*p*^1^	4.54 (d 8.4 Hz)	3.44	3.62	3.92	3.85	3.85, 4.02	96.4 (164.6 Hz)	71.8	72.6	68.8	73.75	69.3
α-d-Gal*p*^1^	5.22 (d 4.0 Hz)	3.75	3.85	3.98	4.23	3.77, 3.97	92.4 (172.3 Hz)	68.6	68.3	69.3	68.9	69.9
β-d-Gal*p*^2^	4.39 (d 8.4 Hz)	3.48	3.62	3.92	3.85	3.85, 4.02	103.1 (169.7 Hz)	70.7	72.5	68.9	73.83	69.3
β-d-Glc*p*A^3^	4.48 (d 8.0 Hz)	3.30	3.53	3.54	3.70		102.6 (171.0 Hz)	73.2	74.2	79.0	76.1	175.3
α-l-Rha*p*^4^	4.68 (s)	3.88	3.71	3.37	3.97	1.19	100.7 (173.5 Hz)	70.3	70.0	71.9	68.6	16.5

a ^1^H, 400 MHz; ^13^C, 100 MHz. d, doublet.

**FIG 8:**
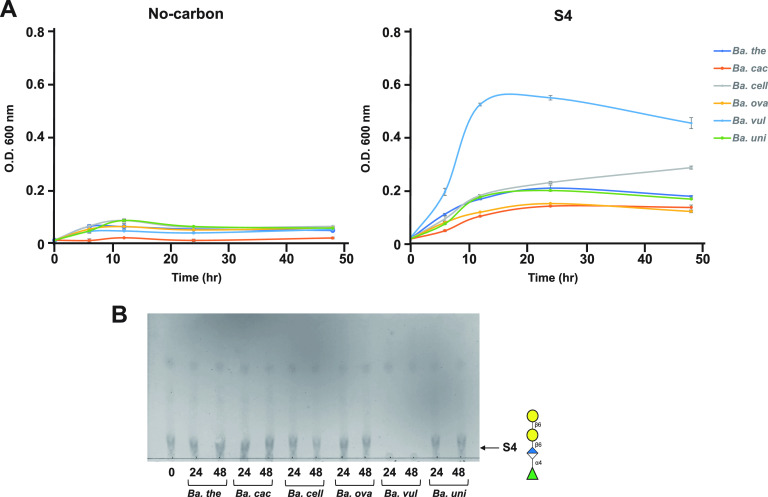
*In vitro* assimilation test of S4 using *Bacteroides* species. (A) Growth of *Bacteroides* species on MM media containing S4 as a sole carbon source (right) and on no-carbon MM medium (left). The absorbance of growth media of Bacteroides thetaiotaomicron (*Ba. the*), Bacteroides caccae (*Ba. cac*), Bacteroides cellulosilyticus (*Ba. cell*), Bacteroides ovatus (*Ba. ova*), Bacteroides vulgatus (*Ba. vul*), and Bacteroides uniformis (*Ba. uni*) was monitored at 0, 6, 12, 24, 48 h (*n *= 3, cell culture biological replicates). Error bars indicate SD (*n *= 3). (B) TLC analysis of the residual S4 after *Bacteroides* species culture at 24 h (24) and 48 h (48) and initial medium without any strains (0). The arrow indicates the position of S4. Data are representative of three independent experiments; one representative experiment is shown.

It was reported that some *Bacteroides* species encode degradative enzymes for tetrasaccharide S4 in gum arabic AGP (7). Therefore, we performed *in vitro* assimilation tests using some *Bacteroides* species in a medium containing S4 as the sole carbon source. Six *Bacteroides* species, including B. thetaiotaomicron, B. caccae, B. cellulosilyticus, B. ovatus, B. vulgatus, and B. uniformis, were tested. As a result, B. vulgatus was seen to exhibit good growth and S4 utilization (Fig. 8). Although B. vulgatus was reported to be a nonutilizer of intact gum arabic AGP (7), it utilized the limit product generated by B. longum from gum arabic AGP.

## DISCUSSION

In this study, we revealed that the multidomain enzyme BlArafE encoded in B. longum subsp. *longum* JCM1217 is an α-l-arabinofuranosidase that hydrolyzes α1,3/1,4-Ara*f* linkages present on gum arabic AGP side chains. Furthermore, the GH43_22 domain in BlArafE hydrolyzed α1,3-Ara*f* linkages on α1,3- and α1,4-Ara*f* double-substituted structure and on α1,3-Ara*f* single-substituted structure. Conversely, the GH43_34 domain in BlArafE hydrolyzed α1,4-Ara*f* linkages on α1,4-Ara*f* single-substituted structure. For degrading S5-GA, one of the main structural components of gum arabic AGP, the removal of terminal α-d-Gal*p*-(1→3)-l-Ara or Gal on S5-GA is necessary for BlArafE activity. Although GAfase is a crucial enzyme for the removal of α-d-Gal*p*-(1→3)-l-Ara, it was only conserved in 6.84% of the B. longum strains according to the NCBI database (9). As 54.5% of the B. longum strains conserve *BlArafE*, it is likely that there would be an unidentified secretory α-d-galactosidase present in the microbiome.

Among GH43 enzymes, there are some proteins containing multiple GH43 catalytic domains belonging to different subfamilies. The previous study based on the CAZy database (29) showed that 82 GH43 proteins possessing the GH43_22 (first domain from N terminus) and GH 43_34 (second domain from N terminus) domains were found, which is the most common combination pattern among multiple GH43 proteins. In particular, 143 GH43 proteins among 228 multidomain enzymes possess the GH43_34 domain at the C terminus, suggesting a functional role of this domain (29). Nevertheless, only four proteins have been characterized among the GH43_34-containing proteins, β-d-galactofuranosidase (Bovatus_03644) from B. ovatus ATCC 8483 (30) and *Paenibacillus* sp. JDR-2 (Pjdr2_0435) (30) and two α-l-arabinofuranosidases (BT_3675 and BT_3662) from B. thetaiotaomicron VPI-5482, based on the CAZy database. BT_3675 acts on the α1,3-Ara*f* linkage present in single α1,3-Ara*f*-substituted gum arabic AGP (7), and BT_3662 acts on α1,3-Ara*f* linkage present in grape RG-II (22). However, in this study, the GH43_34 domain in BlArafE exhibited activity toward α1,4-Ara*f* linkage but not toward α1,3-Ara*f* in gum arabic AGP. Among GH43_22-containing proteins, four proteins have been characterized, β-xylosidase (BAD_1527) from B. adolescentis ATCC 15703 (25), two α-l-arabinofuranosidases (BlArafB and BlArafA) from B. longum subsp. *longum* JCM1217, and arabinanase 43A (RjAbn43A) from *R. josui* JCM17888. Based on a previous study, BlArafB acts on the α1,5-Ara*f* linkage of arabinan, and BlArafA acts on the α1,3-Ara*f* linkage of AGPs (12). Recently, it was found that GH43_22 in BlArafE acts on α1,3-Ara*f* single-substituted arabinoxylan (M. Komeno et al., unpublished data), and RjAbn43A also acts on α1,3-Ara*f* linkage on branched arabinan (24). In the present study, we found that GH43_22 and GH43_34 catalytic domains of BlArafE performed a sequential α1,3- and α1,4-dearabinosylation of the double-substituted side chains of arabinosyl present in gum arabic AGP. Further, the GH43_34 domain of BlArafE was the first example of an α1,4-Ara*f*-specific α-l-arabinofuranosidase. Similar to BlArafE, some multicatalytic enzymes possessing multiple catalytic domains on the same polypeptide have been characterized so far. For example, CelA from Caldicellulosiruptor bescii (31) and ChiA from Flavobacterium johnsoniae (32) possess exo- and endo-acting enzyme catalytic domains and exhibit intramolecular synergy for degrading recalcitrant crystalline substrates. These enzymes show higher activity when they form multicatalytic enzymes than when their equivalent single catalytic domains are added. Regarding soluble substrates, the multicatalytic enzyme Abf43A-Abf43B-Abf43C (GH43_22_26_34) from R. josui was shown to possess GH43_22 (Abf43A) and GH43_26 (Abf43B), which cooperatively degraded sugar beet arabinan (24). However, there are no studies reporting catalytic improvement due to the coexistence of multicatalytic domains in a single polypeptide. In the case of BlArafE, the coexistence of GH43_22 and GH43_34 catalytic domains in a single polypeptide appears to increase the encounter rate with substrates and improve the enzymatic activity considering that GH43_34 cannot act unless trimming is performed by GH43_22 or GAfase. Nevertheless, further investigation comparing the activity between the full-length enzyme and the truncated single catalytic domain variants is warranted to assess the advantages of multicatalytic enzymes for soluble substrates.

**FIG 9:**
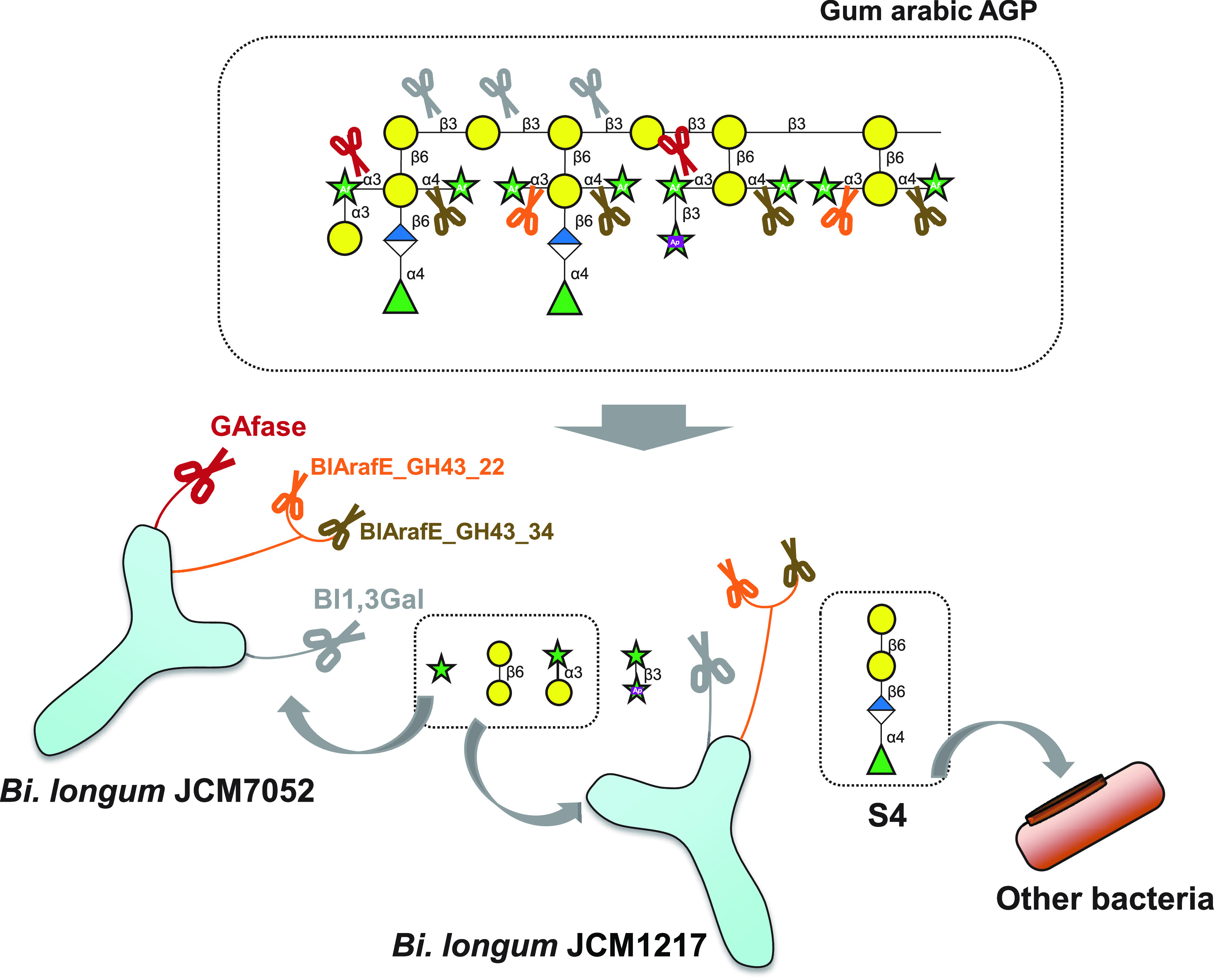
Schematic model of the cooperative degradation of gum arabic AGP by cell surface enzymes in bifidobacteria.

When it became clear that BlArafE was an α1,3/1,4-Ara*f*-specific α-l-arabinofuranosidase, it was revealed that B. longum cooperatively uses three bacterial surface enzymes to degrade gum arabic AGP (Fig. 9). First, α-d-Gal*p*-(1→3)-l-Ara was removed from the end of the side chains by GAfase (BLGA_00340), thereby facilitating the action of BlArafE (BLLJ_1850, BLGA_18450), which is widely conserved in B. longum strains. Furthermore, the trimming of modified sugars in the side chains facilitated the action of Bl1,3Gal (BLLJ_1840, BLGA_18370), finally leading to the accumulation of the tetrasaccharide α-l-Rha*p*-(1→4)-β-d-Glc*p*A-(1→6)-β-d-Gal*p*-(1→6)-d-Gal, which could not be degraded by B. longum. The reaction products produced by recombinant enzymes were consistent with those produced using B. longum subsp. *longum* JCM7052 bacterial cell fraction, suggesting that these enzymes were used for the assimilation of gum arabic AGP by B. longum. However, the β1,3-galactan main chain of gum arabic AGP was not completely degraded by B. longum enzymes, indicating that B. longum favors the metabolism of the modified sugar of the side chain such as Ara and α-d-Gal*p*-(1→3)-l-Ara. This assimilative strategy differs from that of *Bacteroides*, which favors the metabolism of high-degree-of-polymerization (DP) oligosaccharides by cell surface endo-β1,3-galactanase and many accessory enzymes localized in the periplasm (7). However, unlike other colonic *Bacteroides*, B. vulgatus is not a versatile utilizer of polysaccharides, and it seems to favor the metabolism of oligosaccharides generated by other bacteria from polysaccharides as per a previous study in which various polysaccharides, including inulin, levan, amylopectin, and gum arabic AGP, were used (7, 33, 34). In this study, B. vulgatus exhibited good growth in the limit product S4 generated by B. longum subsp. *longum* JCM7052 from gum arabic AGP. pH-controlled batch culture studies and administration tests of gum arabic AGP on human volunteers had shown that the intake of gum arabic AGP increases *Bifidobacterium* and *Bacteroides* (3, 35) and *Bacteroidetes* phyla (36) in the human intestine or the simulation model. Although they could grow on gum arabic AGP using the assimilative strategy described above, cross-feeding may occur between bifidobacteria and *Bacteroides* species. Further studies using cocultures or pH-controlled batch cultures in the presence of key enzymes, such as GAfase, BlArafE, Bl1,3Gal, and endo-β1,3-galactanase, are warranted to investigate the symbiosis between bifidobacteria and *Bacteroides* in terms of the assimilation of gum arabic AGP.

## MATERIALS AND METHODS

### Materials.

Gum arabic (Acacia senegal) was obtained from Sigma-Aldrich (St. Louis, MO, USA). Larch AGP was purchased from Tokyo Chemical Industry Co., Ltd. (Tokyo, Japan). Gum arabic AGP and Larch AGP were used after purification using ethanol precipitation. α-d-Gal*p*-(1→3)-l-Ara-free gum arabic AGP was prepared based on the method described in our previous report (9). Gum arabic AGP (1%) was incubated with GAfase at 37°C in a dialysis membrane (size 36; Wako Pure Chemical Industries Ltd., Osaka, Japan) and dialyzed against 50 mM sodium acetate buffer (pH 5.5). The polysaccharide was then precipitated with 80% ethanol. All other chemicals used in the study were obtained from Wako Pure Chemical Industries Ltd. (Osaka, Japan).

### Preparation of gum arabic AGP-derived oligosaccharides.

The schematic structures of oligosaccharides and brief purification process are shown in Fig. 1 and Fig. S2. S5-GA, S4-GA, and S3-GA were prepared from the supernatant obtained from ethanol precipitation of gum arabic as described previously (8, 9). Briefly, the ethanol supernatants were dried, dissolved in water, and separated via gel filtration chromatography on a Bio-Gel P-2 column (dimensions [φ], 25 by 830 mm; Bio-Rad Laboratories, Hercules, CA, USA). Subsequently, the oligosaccharides were purified via using an activated carbon column (Autoprep FiberAC; Showa Denko, Tokyo, Japan), high-pressure liquid chromatography on a Cosmosil Sugar-D column (φ, 4.6 by 250 mm; Nacalai Tesque Inc., Kyoto, Japan), and a Cosmosil PBr column (φ, 4.6 by 250 mm; Nacalai Tesque Inc.). The structures of S5-GA, S4-GA, and S3-GA were confirmed by enzymatic reaction and MS analysis. S5 and S3 were prepared from 0.01 mM S5-GA and 0.05 mM S3-GA by enzymatic reaction with GAfase (2.9 nM and 58 nM, respectively) at 37°C until the reactions were completed. S5-A, S4-A, and S3-A were prepared from 0.01 mM S5-GA, S4-GA, and S3-GA by enzymatic reaction at 37°C with GH36 α-d-galactosidase BlAga3 from B. longum subsp. *longum* JCM7052 (0.10 μM) (5). The reaction mixture was loaded onto a graphitized carbon (GC) cartridge column (InertSep GC column, 2 g/12 mL; GL Sciences, Tokyo, Japan) for further cleanup and enrichment of S5-A and S5. GC cartridge columns were equilibrated with 10 mL of 0.1% trifluoroacetic acid in 80% acetonitrile and 8 mL of water before sample loading. After loading the samples, oligosaccharides were eluted in 80% (vol/vol) acetonitrile containing 0.5% (vol/vol) trifluoroacetic acid. The dried samples were dissolved using an appropriate amount of water and used as the substrate.

### Expression and purification of recombinant BlArafE.

The genomic DNA of B. longum subsp. *longum* JCM1217 (GenBank accession no. AP010888.1) was used for PCR amplification of the gene encoding BLLJ_1850 (BlArafE; GenPept accession no. BAJ67514) as a template. The forward (BLLJ_1850_for) and reverse (BLLJ_1850_rev) primers (Table 1) were designed for amplifying the 88 to 4899 residues of *BlArafE* without the coding sequences of the N-terminal signal peptide (aa 1 to 26) and C-terminal transmembrane region (aa 1637 to 1659). *BlArafE* was amplified by high-fidelity PCR using KOD plus ver. 2 (Toyobo, Japan), and the amplified fragment was ligated into the EcoRI and XhoI sites of the pET23b(+) vector (Novagen, USA). The resulting plasmid was used to transform Escherichia coli BL21 (λDE3) cells (Genlantis, San Diego, CA, USA), which were then cultured at 25°C using the Overnight Express autoinduction system (Novagen). The cell culture was centrifuged, and the resultant pellet was resuspended in the BugBuster protein-extraction reagent (Novagen). N-terminal His-tagged BlArafE protein was purified using a column containing Talon metal-affinity resin (Clontech Laboratories Inc., Palo Alto, CA, USA). The purified fraction was desalted and concentrated using an ultrafiltration membrane (30-kDa molecular weight cutoff; Millipore Co., Billerica, MA, USA). The recombinant BlArafA, BlArafB, GAfase, and Bl1,3Gal enzymes used in this study were prepared according to previous studies (11, 12).

### Enzyme assays.

The reactivity of the recombinant enzymes was analyzed using the following substrates: polysaccharides [gum arabic AGP, larch AGP, and α-d-Gal*p*-(1→3)-l-Ara-free gum arabic AGP] and gum arabic AGP-related oligosaccharides (S5-GA, S5-A, S5, S4-A, S3-GA, S3-A, and S3). In the case of the polysaccharides, each substrate (final concentration, 1.0%) was incubated with 2.2 nM BlArafA, 2.2 nM BlArafB, or 1.4 nM BlArafE in 40 μL of 50 mM sodium acetate buffer (pH 6.0) for 16 h. For the gum arabic AGP-related oligosaccharides, each substrate (final concentration, 0.01 mM) was incubated with 0.88 nM BlArafA, 0.90 nM BlArafB, or 0.57 nM BlArafE in 100 μL of 50 mM sodium acetate buffer (pH 6.0) for 16 h. The reaction products were analyzed by TLC or HPAEC-PAD. In the TLC analysis, the reaction products were spotted on a silica gel 60 aluminum plate (Merck, Darmstadt, Germany) with a 7:1:2 (vol/vol/vol) 1-propanol/ethanol/water solvent mixture, and the separated sugars were visualized by spraying an orcinol-sulfate reagent on the plates. In HPAEC-PAD analysis, a CarboPac PA-1 column (φ, 4 by 250 mm; Dionex Corp., Sunnyvale, CA, USA) was used with the flow rate of 1.0 mL/min using the following gradient: 0 to 5 min, 100% eluent A (0.1 M NaOH), 5 to 30 min, 0% to 100% eluent B (0.5 M sodium acetate in 0.1 M NaOH), and 30 to 35 min, 100% eluent B.

### Site-directed mutagenesis.

A KOD Plus mutagenesis kit (Toyobo Co., Ltd., Osaka, Japan) was used to introduce D571A and D1197A amino acid substitutions into BlArafE using specific primers (Table 1). The nucleotide sequences were confirmed by sequencing. BlArafE_D571A and BlArafE_D1197A mutants were expressed and purified using the same method as that used for the wild-type BlArafE. Enzyme assay of the mutant enzymes was performed using the gum arabic AGP-related oligosaccharides (S5-GA, S5-A, S5, and S4-A) and polysaccharides [gum arabic AGP, α-d-Gal*p*-(1→3)-l-Ara-free gum arabic AGP, and larch AGP]. For gum arabic AGP-related oligosaccharides, S5-GA, S5-A, and S5 (final concentration, 0.025 mM) were incubated with 0.80 nM BlArafE_WT, 1.1 nM BlArafE_D571A, and 0.47 nM BlArafE_D1197A in 100 μL of 50 mM sodium acetate buffer (pH 6.0) for 16 h. S4-A (final concentration, 0.024 mM) was incubated with 2.1 nM BlArafE_WT, 2.9 nM BlArafE_D571A, or 1.2 nM BlArafE_D1197A in 20 μL of 50 mM sodium acetate buffer (pH 6.0) for 16 h. Polysaccharides (final concentration, 1.0%) were incubated with 1.0 nM BlArafE_WT, 1.4 nM BlArafE_D571A, and 0.57 nM BlArafE_D1197A in 40 μL of 50 mM sodium acetate buffer (pH 6.0) for 16 h. The reaction products were analyzed by TLC or HPAEC-PAD, as mentioned above.

### *In vitro* assimilation test of α-d-Gal*p*-(1→3)-l-Ara-free gum arabic AGP using B. longum strains.

The following four strains of B. longum used in *in vitro* assimilation tests were obtained from stock cultures maintained at the Morinaga Milk Industry Co., Ltd., Zama, Japan: MCC00300, MCC00055, MCC00198, and MCC00231. B. longum subsp. *longum* JCM7052 and JCM1217 were obtained from the Japan Collection of Microorganisms (Riken Bioresource Center, Ibaraki, Japan). The strains were precultured at 37°C under anaerobic conditions in MRS broth containing 0.05% l-cysteine hydrochloride using an AnaeroPack system (Mitsubishi Gas Chemical, Tokyo, Japan). The precultured cells were inoculated into MRS medium containing 1.0% gum arabic AGP or α-d-Gal*p*-(1→3)-l-Ara-free gum arabic AGP as the sole carbon source and then cultured for 48 h at 37°C under anaerobic conditions. The absorbance at 600 nm of each culture medium was measured after 8, 12, 23, and 48 h. The experiment was performed in duplicates. To analyze the residual l-arabinose on α-d-Gal*p*-(1→3)-l-Ara-free gum arabic AGP after culture, the polysaccharide fraction was separated using ethanol precipitation. The precipitate was dissolved in water and then incubated with 0.021 μM BlArafE in 40 μL of 50 mM sodium acetate buffer at 37°C for 16 h. The reaction products were analyzed by TLC, as mentioned above.

### Combination reactions of type II AG degradative enzymes.

Gum arabic AGP (0.25%) was incubated with different combinations of 20 nM GAfase, Bl1,3Gal, and BlArafE, and BlArafA in 40 μL of 50 mM sodium acetate buffer (pH 6.0) at 37°C for 24 h. The reaction products were analyzed by HPAEC-PAD as described above. Data from one representative experiment of three independent experiments are shown. To assay bacterial enzyme activity, the freeze-thawed cell pellets of B. longum subsp. *longum* JCM1217 and JCM7052 grown on larch AGP were incubated with 1.0% gum arabic AGP in 50 mM sodium acetate buffer at 37°C for 16 h, and the reaction products were analyzed using HPAEC-PAD as described above.

### Structural identification of oligosaccharide S4.

The oligosaccharide S4 was obtained from gum arabic AGP (final concentration, 5.0%) following incubation with the bacterial cell fraction of B. longum subsp. *longum* JCM7052 grown in larch AGP in 400 mL of 50 mM sodium acetate buffer (pH 5.0) for 53 h. The released oligosaccharides were obtained by ethanol precipitation, and the supernatant was evaporated to dryness. S4 was separated via gel-filtration chromatography on a Bio-Gel P-2 column (φ, 25 by 830 mm; Bio-Rad Laboratories, Hercules, CA, USA) equilibrated with water and high-pressure liquid chromatography using a Cosmosil PBr column (φ, 4.6 by 250 mm; Nacalai Tesque Inc.) and was eluted using 20 mM sodium phosphate (pH 2.5). Finally, the sample was desalted by gel filtration chromatography on a Bio-Gel P-2 column equilibrated with water. The freeze-dried sample was dissolved in deuterium oxide (D_2_O). ^1^H and ^13^C NMR spectra and two-dimensional spectra (^1^H-^1^H COSY, HMQC with/without ^13^C-^1^H decoupling, HMBC) were measured in D_2_O at room temperature on a Jeol ECX 400 spectrometer (400 MHz). For MS, MALDI-TOF mass spectra were recorded on Shimadzu Kompact MALDI Axima-CFR spectrometer with 2,5-dihydroxybenzoic acid as the matrix. ESI-TOF mass spectra were recorded on Jeol AccuTOF JMS-T700LCK with CF_3_CO_2_Na as the internal standard.

### *In vitro* assimilation test of *Bacteroides* species on S4.

The following six *Bacteroides* species used in *in vitro* assimilation tests were obtained from the Japan Collection of Microorganisms: B. thetaiotaomicron JCM5827, B. caccae JCM9498, B. cellulosilyticus JCM15632, B. ovatus JCM5824, B. vulgatus JCM5826, and B. uniformis JCM5828. The strains were precultured in Gifu anaerobic modified medium (Nissui Pharmaceutical, Japan) at 37°C under anaerobic conditions. The precultured cells were inoculated into minimal medium (MM) (37) containing S4 as a sole carbon source (final concentration of 0.5% [wt/vol]) and then cultured at 37°C under anaerobic conditions. MM contained 100 mM KH_2_PO_4_, 15 mM NaCl, 8.5 mM (NH_4_)_2_SO_4_, 4 mM l-cysteine, 200 μM l-histidine, 1.9 μM hematin, 100 μM MgCl_2_, 1.4 μM FeSO_4_·7H_2_O, 50 μM CaCl_2_, 1 μg/mL of vitamin K_3_, and 5 ng/mL of vitamin B_12_. The absorbance at 600 nm was measured after 0, 6, 12, 24, and 48 h. The experiment was performed in triplicates. The supernatants were analyzed by TLC as mentioned above to analyze the residual S4 after culturing for 24 and 48 h.
