# Supplementary material for: Mechanism of Cooperative Degradation of Gum Arabic Arabinogalactan Protein by Bifidobacterium longum Surface Enzymes
Source: Appl Environ Microbiol. 2022 Mar 22;88(6):e02187-21. doi: 10.1128/aem.02187-21 (PMC8939339; doi:10.1128/aem.02187-21)
Supplement: Supplemental file 1 — Fig. S1 to S5. Download aem.02187-21-s0001.pdf, PDF file, 5.2 MB [file aem.02187-21-s0001.pdf]

Supplementary Figure Legends

Supplementary Fig. S1. SDS-PAGE analysis of recombinant BlArafE. Purified BlArafE WT was electrophoresed on a 5–20% gradient polyacrylamide gel and stained with Coomassie Brilliant Blue R-250. Lane 1, molecular size marker; lane 2, purified BlArafE_WT. Arrow indicate target protein at expected molecular size.

Supplementary Fig. S2. HPAEC-PAD analysis of the oligosaccharides as substrates used in this study and the brief purification process.

Supplementary Fig. S3. HPAEC-PAD analysis of the reaction products by α-L-arabinofuranosidases with gum arabic AGP-related oligosaccharides. S5-GA, S5-A, S5, S4-A, S3-GA, S3-A, and S3 were incubated with either BlArafE, BlArafB, or BlArafA at 37°C for 16 h.

Supplementary Fig. S4. The schematic model of action of BlArafE and BlArafA on gum arabic AGP and α-D-Gal*p*-(1→3)-L-Ara-free gum arabic AGP.

Supplementary Fig. S5. MS analysis (A) and NMR analysis (B) of S4.

(A) Mass spectrometry of S4 and the estimated structure are shown in the figure. (B) 2D-NMR spectra were measured on 400 MHz; ^{1}H-^{1}H COSY at 22°C (a), HMQC at 21°C (b), HMBC at 21°C (c), and non-decoupled HMQC at 21°C (d).

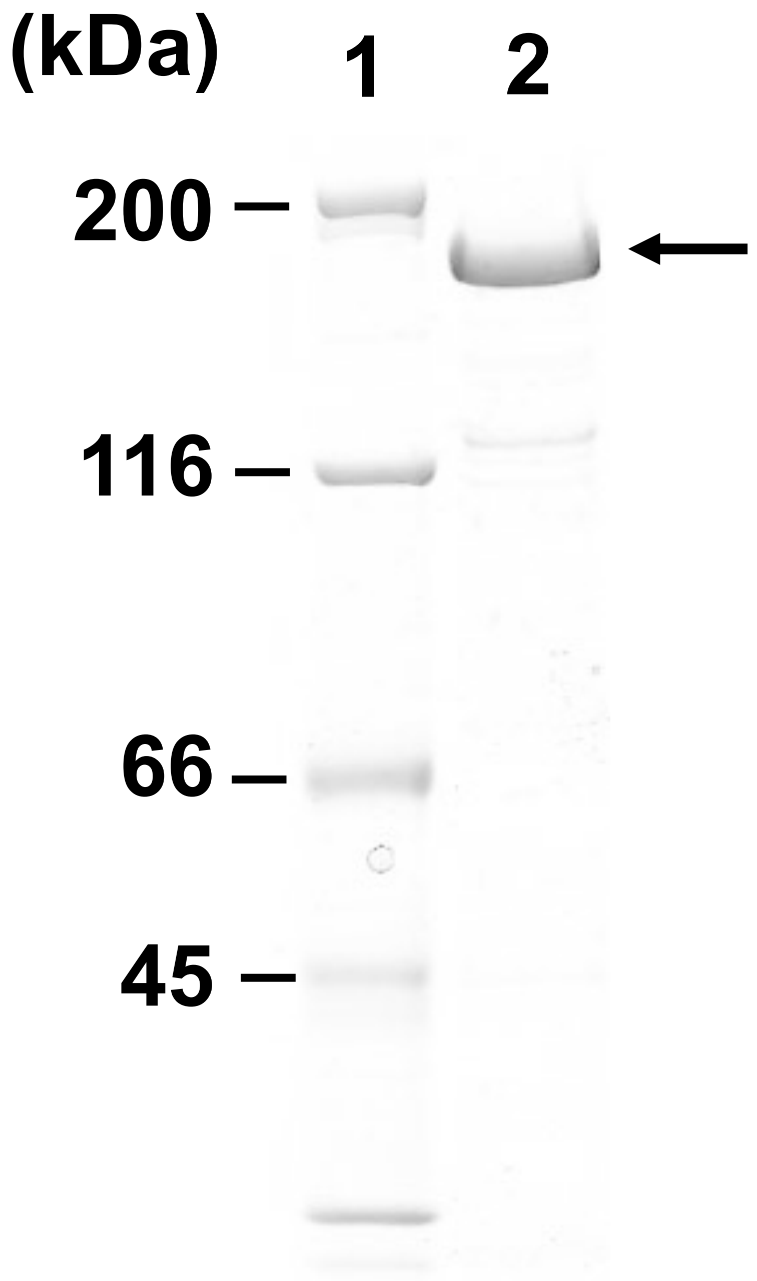

Supplementary Fig. S1, Sasaki et al.

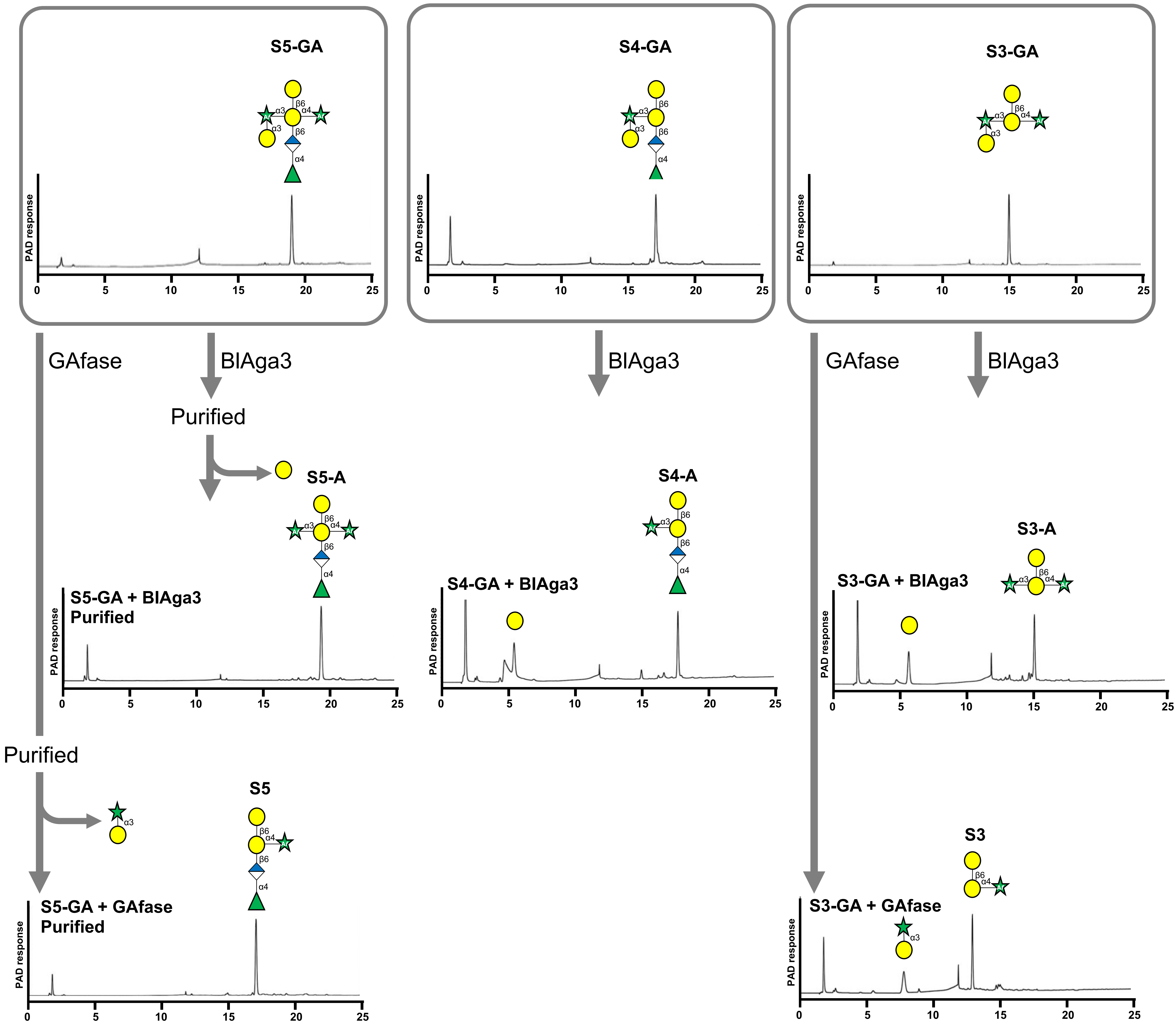

Supplementary Fig. S2, Sasaki et al.

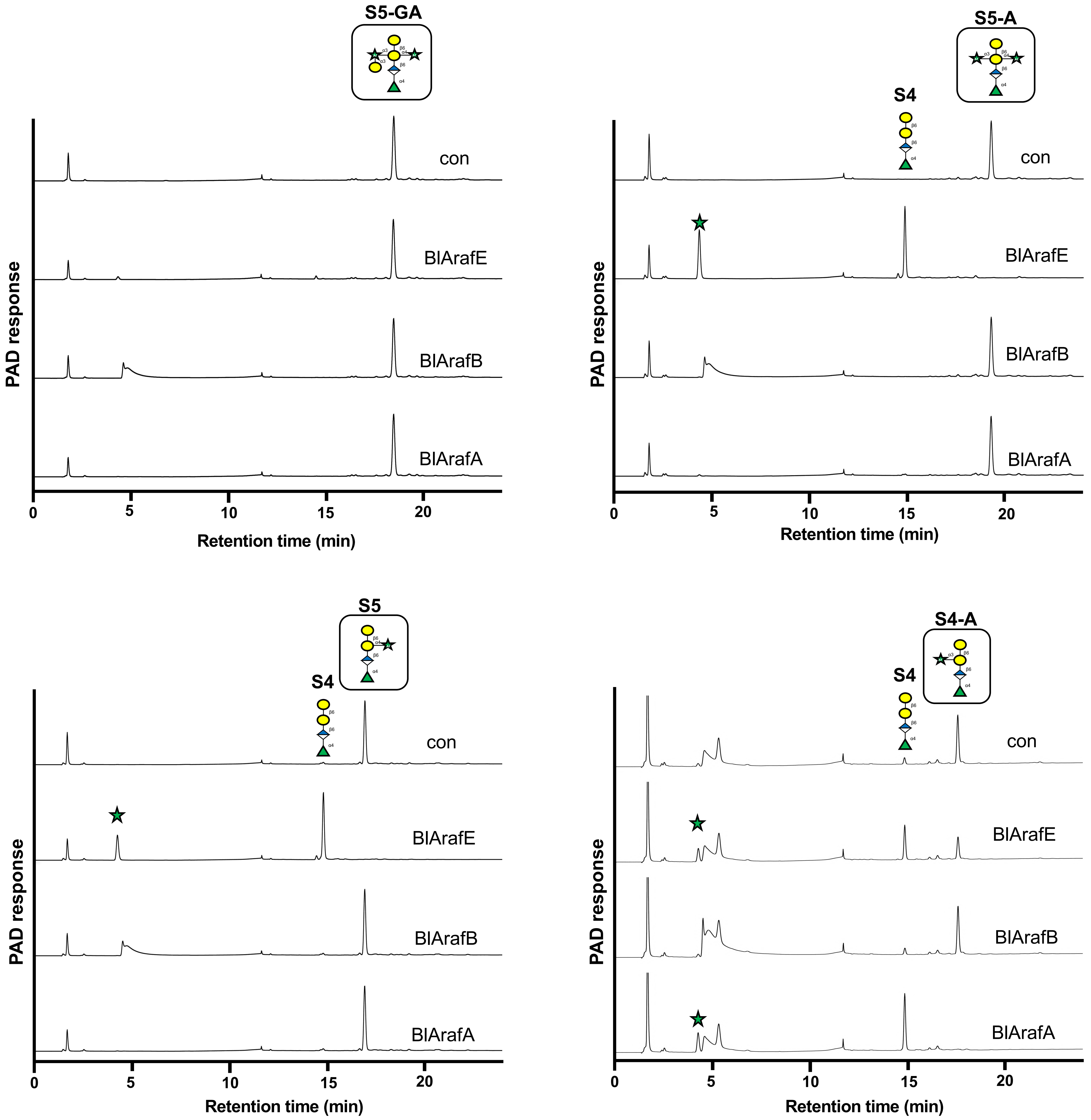

Supplementary Fig. S3, Sasaki et al.

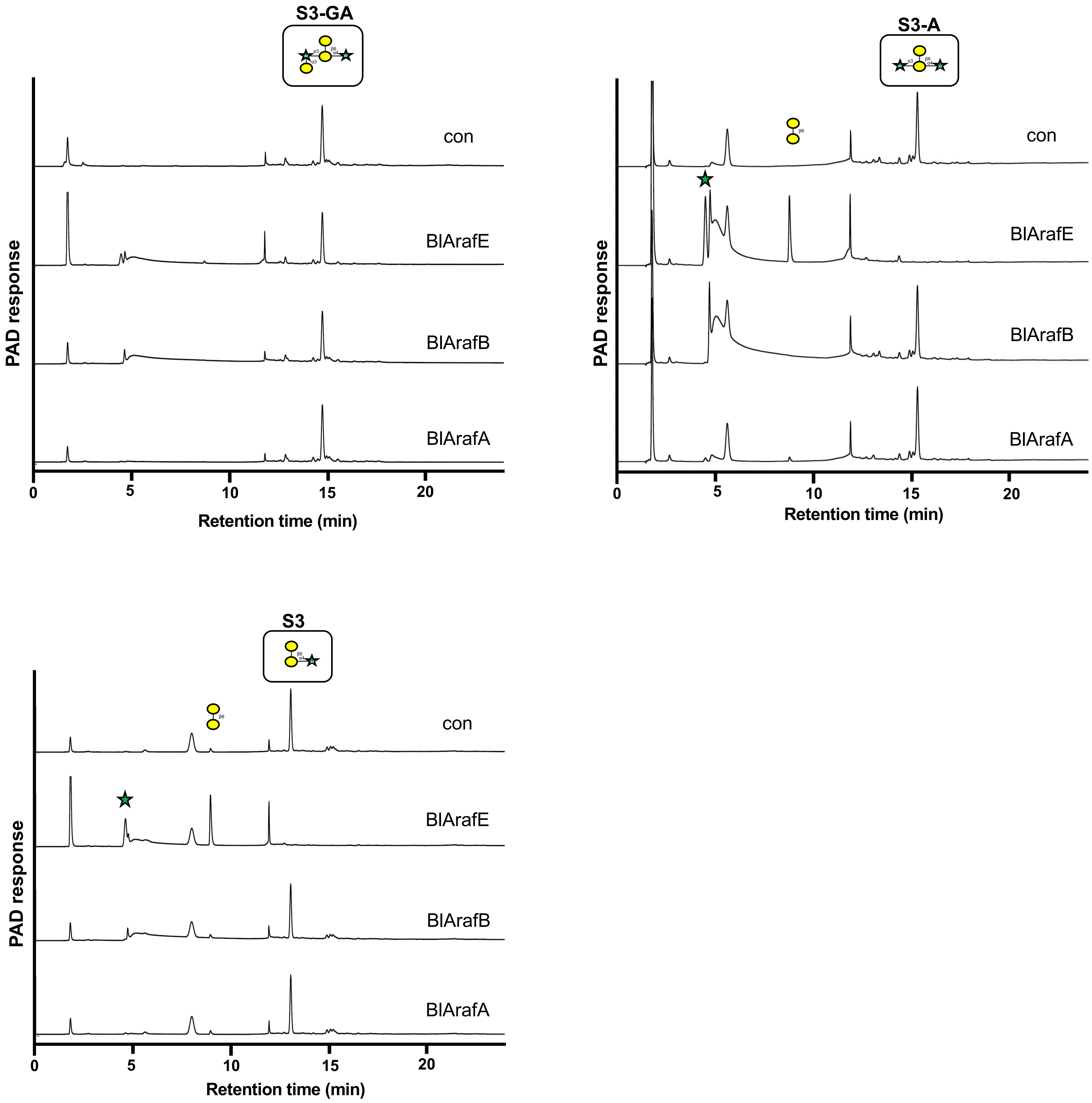

Supplementary Fig. S3, Sasaki et al. (Continued)

Gum arabic AGP

α–D-Gal*p*-(1→3)-L-Ara-free gum arabic AGP

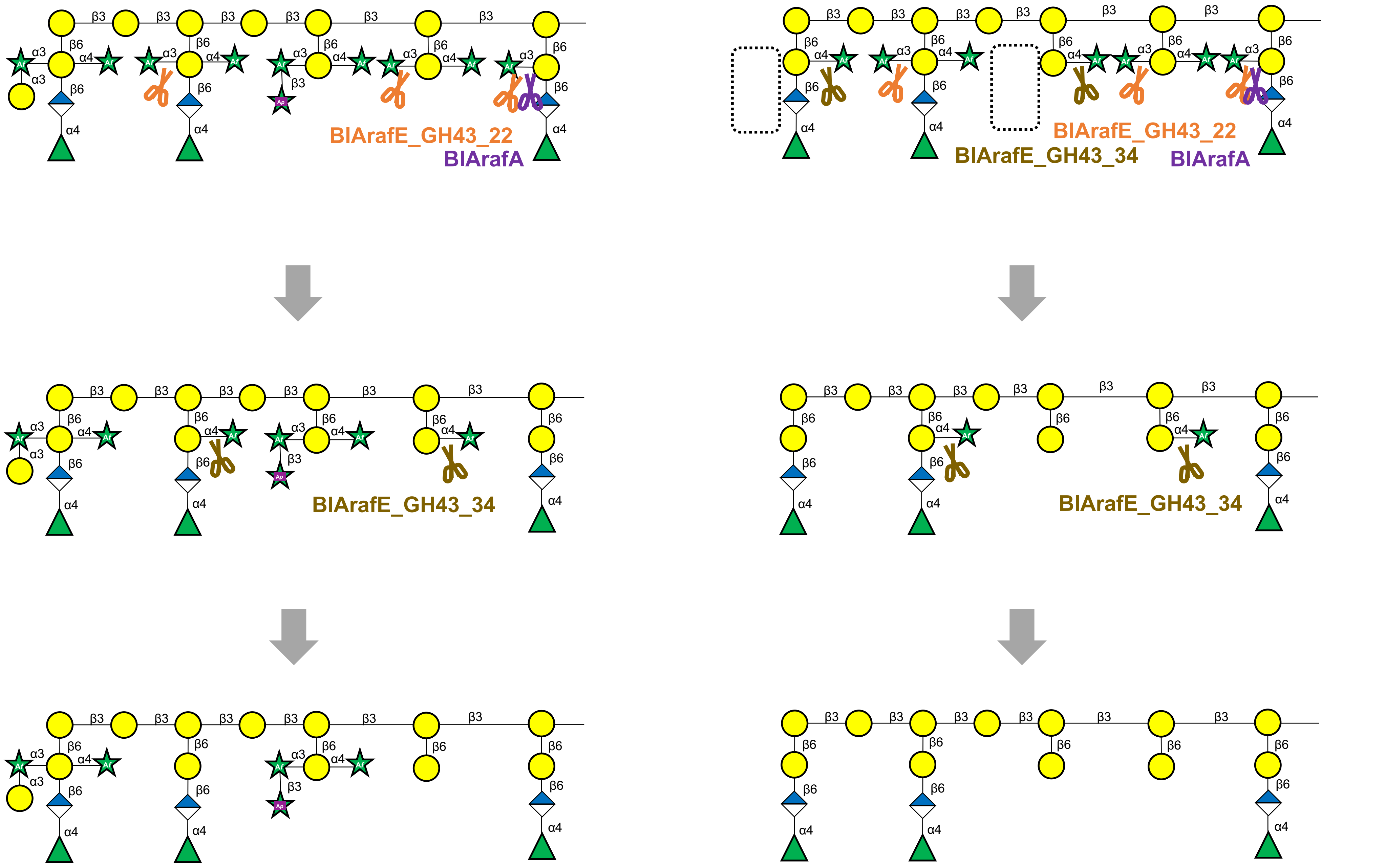

Supplementary Fig. S4, Sasaki et al.

A

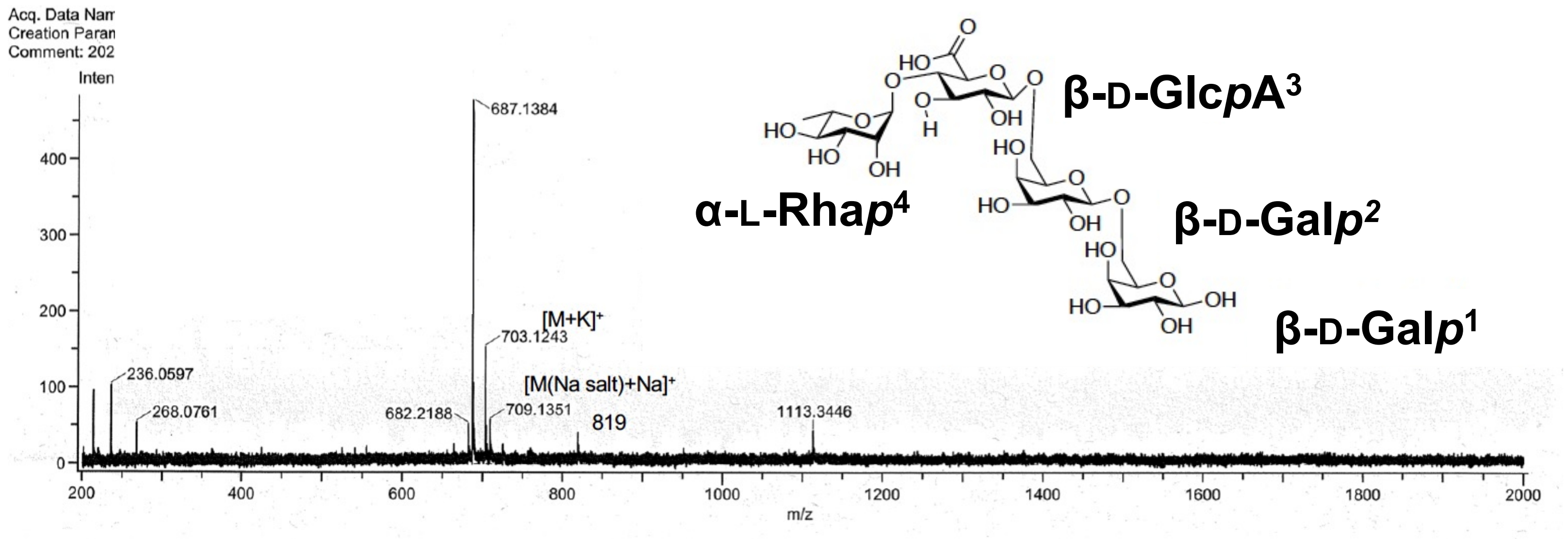

B

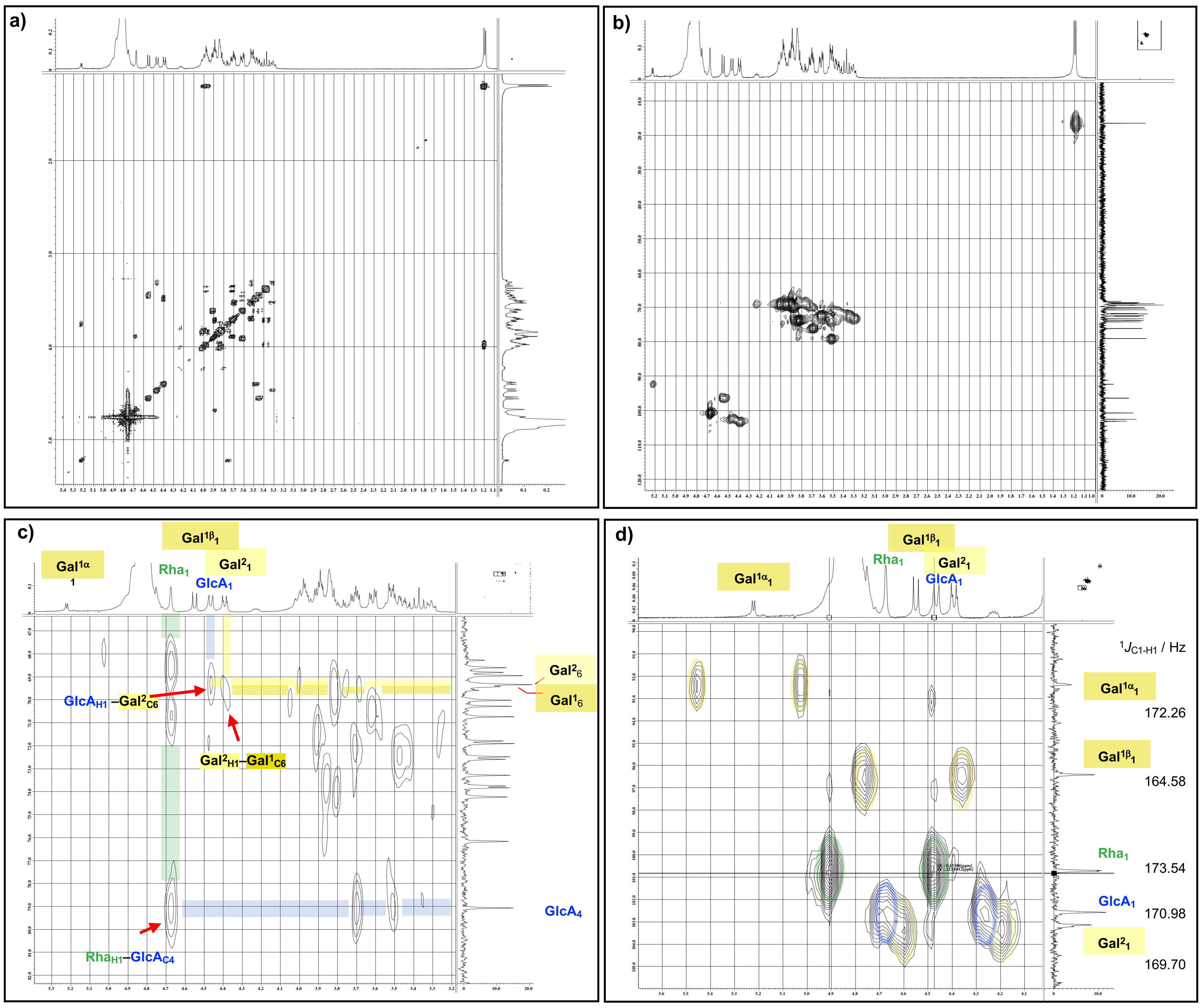

Supplementary Fig. S5, Sasaki et al.
